# Dispersion Mechanism of Styrene–Butadiene Rubber Powder Modified by Itaconic Acid and Its Toughening Effect on Oil Well Cement

**DOI:** 10.3390/ma15238345

**Published:** 2022-11-23

**Authors:** Yubing Xing, Miaomiao Hu, Chengzhang Cao, Jiayu Yu, Jiaqi Zhao, Hongbing Zheng, Jintang Guo

**Affiliations:** 1 Department of Polymer Science and Engineering, School of Chemical Engineering and Technology, Tianjin University, Tianjin 300354, China; 2 Zhejiang Shaoxing Institute of Tianjin University, Shaoxing 312300, China; 3 Drilling Technology Research Institute, Sinopec Shengli Oilfield Service Corporation, Dongying 257000, China; 4 Shandong Chambroad Sinopoly New Materials Co., Ltd., Binzhou 256600, China

**Keywords:** SBR latex powder, dispersion state, cement hydration, retardation, toughness

## Abstract

Styrene–butadiene rubber (SBR) has been extensively applied to enhance the toughness of hardened cement. The instability of existing liquid latex leads to difficulties in storage and transportation, and even performance regression. Thus, the well-dispersed carboxylated butylbenzene (SISBR) latex powders were fabricated through the seed emulsion polymerization of liquid polybutadiene (LPB), styrene (St), itaconic acid (IA), and sodium p-styrenesulfonate (SSS) to overcome the difficulties. The dispersion performance of latex powders with various IA amounts was quantitatively evaluated using particle size distribution, zeta potential, and ultraviolet–visible spectrophotometry. Results showed that the carboxylic ionic (COO-) from IA enhanced the dispersing abilities of SISBR latex powders, which ensured the uniform distribution in water. Based on this, the influence of latex powder on cement was assessed mainly by fluidity, isothermal heat flow calorimetry, X-ray diffraction (XRD), and triaxial mechanical testing. Results showed the fluidity and dispersion performance of cement were improved with more IA in latex, while the hydration of cement was retarded due to excessive adsorption of carboxyl (-COOH) groups in IA. Triaxial mechanical testing showed that cement with SISBR-3 (latex containing 3% IA) exhibited the minimal elastic modulus of 3.16 GPa, which was lower than that of plain cement (8.34 GPa).

## 1. Introduction

As the primary aim of cementing, efficient zonal isolation determines the safe exploration of oil and gas [1]. Under extreme conditions, such as hydraulic fracturing, large temperature difference, and high pressure, poor bonding and cracks in the cement sheath will lead to fluctuations of the sustained casing pressure and migration of formation fluids, imperiling the integrity and service life of the well [2]. Since the brittleness of cementitious materials make them prone to cracking and debonding, the toughness and deformation ability of cement sheaths are important to their seal-integrity. Typically, toughening materials include steel fibers [3,4], polymer latex [5], rubber particles [6], and nanomaterials, etc. Among them, polymer latex is most widely applicable for oil and gas cementing engineering, due to its key ability to form uniform and flexible latex films, which exhibt good cohesion and adhesion in cement and concrete compared with other toughening materials.

Styrene–butadiene rubber (SBR), currently one of the most widely used latex materials, has been extensively adopted to enhance the toughness of hardened cement through filling pores in cement and dissipating external mechanical force. To meet the demand for latex in cementing under complicated conditions, various modifications of latex have been used. The modified latex grains are adsorbed onto the surface of cement particles due to the functional groups (e.g., sulfonic acid, carboxylic acid, and amide), affecting the properties exhibited by cement. Plank et al. [5] compared the adsorption abilities of cationic and anionic latex grains by conducting the adsorption and zeta potential measurements. On that basis, the adsorption ability of anionic latex grains on cement grains was verified to be stronger than that of cationic latex grains. Fan et al. [7] demonstrated that latex modified by carboxylic groups exhibited stronger adsorption capacity on cement particles than sulfonic groups, which also resulted in greater improvement in the toughness of the cement. Wang et al. [8] found carboxylated styrene–butadiene latex (XSBR) film not only physically filled cement pores, but also reacted with hydrated products to form a 3D network due to carboxylic groups, which exhibited superior performance compared with SBR. To some extent, the presence of liquid latex addresses the brittleness of cementitious material. However, the previously reported liquid latex [7] resulted in a dynamically unstable system, and the stability decreased with storage time, resulting in shortened shelf-life. The instability largely up-regulated its transportation and storage costs, even impairing the toughening efficiency. Meanwhile, the leakage of liquid latex usually causes environmental pollution during application and storage [9,10]. The dispersion medium of latex, water, should be reduced or avoided. The transformation from liquid latex to the dry powder through spray-drying, which can be redispersed is the most economical way.

The spray-drying process could potentially contribute to the instability of emulsion through altering the interfacial properties. Hence, the key point is to maintain the uniformity of the powder in water after spray-drying. Kim et al. introduced lignin to the drying process of nanocellulose to settle the nonreciprocal agglomeration and consequent regression at the physicochemical properties [11]. He et al. used gelatin to form a protective film wrapped on the surface of nanocellulose, which ensured good stability and dispersion kinetics through steric action [12]. Keerthika examined the role of anchoring ability of butyl/phenyl groups in the block copolymer for providing redispersibility and stability of the latex, by comparison with surfactants devoid of anchoring units [13]. In addition to assisting dispersion with admixtures, it has also been reported that carbon nanotubes were modified with butadiene according to the anion-based functionalization method to achieve good dispersion [14].

To resolve the transportation and storage difficulties during engineering application, it is urgent to find a latex powder with good dispersion properties to toughen cement. Herein, the well-dispersed carboxylated butylbenzene (SISBR) latex powder was prepared by introducing itaconic acid (IA) in SBR, the effects of IA on dispersion state and toughening efficiency of latex powder were comprehensively investigated. The dispersion states of latex powders with various IA amounts were analyzed by zeta potential, absorbance, and particle size. The performance of cement pastes with SISBR latex powders were investigated by fluidity testing. Their effects on hydration kinetics of cement slurry and the reaction mechanism were systematically examined by conducting isothermal calorimetry, X-ray diffraction (XRD) analysis, and total organic carbon (TOC) analysis. Finally, the compressive strength, toughness and microstructure of cement with the latex were measured. The preparation of highly dispersed latex powder is of broad application prospect and good economic benefits [15].

## 2. Experimental Section

### 2.1. Materials

The cement utilized for the study was G class oil well cement purchased from Jiahua Special Cement Co., Ltd. (Sichuan, China). The chemical composition of the cement is showed in Table 1, and it was tested using X-ray fluorescence (XRF). Styrene (St, Mw = 104.15, AR) was purchased from Damao Chemical Reagent (Tianjin, China). Liquid polybutadiene (LPB, M_w_ ≈ 1100 g mol^−1^) was technical grade, which was purchased from Sinopec Yanshan Petrochemical (Beijing, China). Itaconic acid (IA) and sodium p-styrene sulfonate (SSS) were purchased from Dibo Biological Technology Co., Ltd. (Shanghai, China). Sodium hydroxide (pH regulator, AR) was purchased from Yuanli Chemical Tech (Tianjin, China). Ammonium persulfate (APS, initiator, AR) and sodium dodecyl sulfate (SDS, surfactant, AR) were purchased from Fuchen Chemical Reagent Co., Ltd. (Tianjin, China). Commercially available styrene butadiene latex (SBR) was purchased from the Drilling Research Institute in China National Petroleum Corporation (Beijing, China).

### 2.2. Preparation of Three Latex Powders

**Figure 1:**
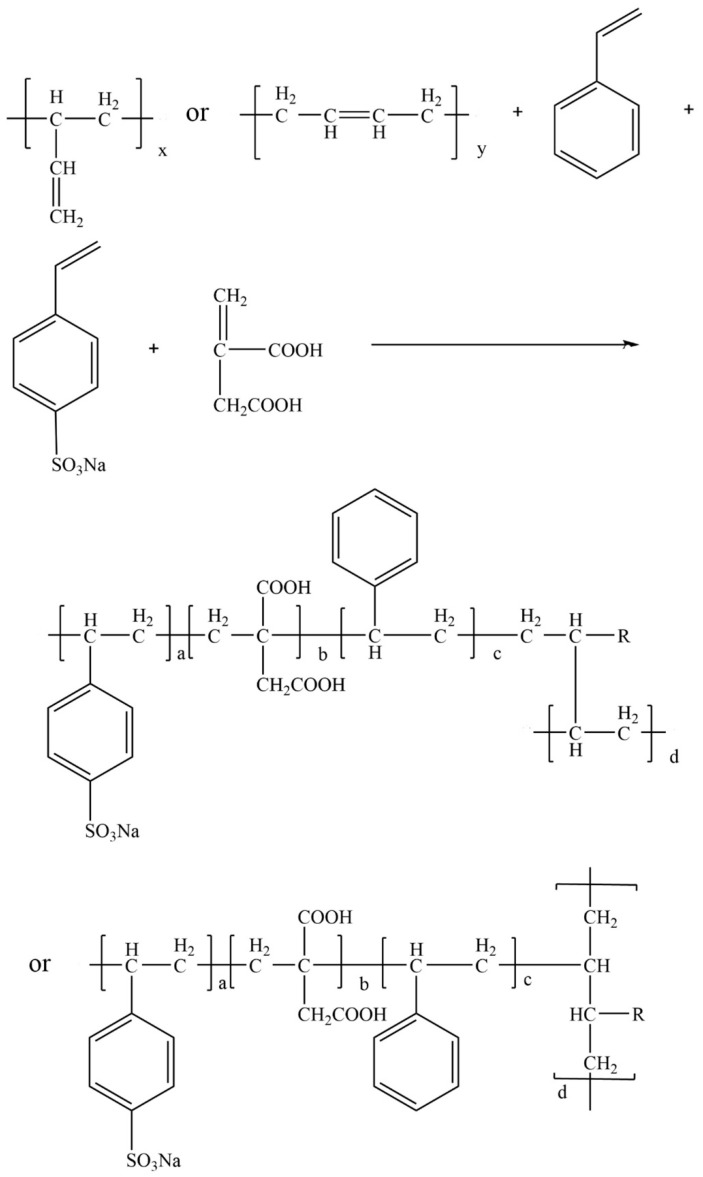
Schematic illustration of synthesis reaction of SISBR latex.

The SISBR was synthesized through seed emulsion polymerization, which used SSS and IA as its functional monomers, providing sulfonate groups and carboxyl groups for the latex, respectively. The synthesis reaction of SISBR latex is shown in Figure 1. To explore the effect of IA on the redispersibility of latex powder, three kinds of latex were prepared using different quantities of IA. The specific synthetic procedure, taking SISBR-1 as an example, can be described as follows. APS initiator and SDS emulsifier accounted for 1% and 2% of the total mass of monomers, respectively. The seed emulsion was prepared by adding part of the styrene monomers to the mixture of a small amount of SSS solution mixed with SDS emulsifier and APS solution, and then reacting for 60 min at 70 °C. The stable monomer solution was obtained by mixing the remaining styrene and polybutadiene, after adding emulsifier in proportion, and treated with ultrasonication. The remaining SSS and IA monomers were dissolved in the aqueous solution and adjusted to be weakly alkaline with Sodium Hydroxide. The monomer emulsion, APS solution, and aqueous phase solution were simultaneously dripped into the vessel containing the seed emulsion with constant rates. The dripping time was controlled at two hours and the temperature was controlled at 70 °C. After the dripping was complete, a constant temperature reaction was carried out for 3 h to obtain a quaternary copolymer SISBR-1 latex. The preparation procedures for SISBR-3 and SISBR-5 were fundamentally the same as above except for the monomer IA dosage. The dosage of monomer IA used to prepare SISBR-3 and SISBR-5 latex was three and five times that for SISBR-1 latex. The three kinds of latex were treated using the spray drying method to obtain latex powder.

### 2.3. Characterization of Latex Powder

#### 2.3.1. Absorbance of the Latex Powder

The change in the absorbance of the suspension with time can be used to characterize the stability of the suspension [16]. The latex powder was configured into aqueous suspension with a solid content of 30%, and then diluted 16,000 times to obtain the sample. The absorbance was determined from a wavelength range of 200 nm to 250 nm with a ultraviolet–visiblespectrophotometer (Shimadzu, Kyoto, Japan).

#### 2.3.2. Particle Size of the Latex Powder

In this part we analyzed the particle size and distribution for SISBR latex powders using a ZetasizerNano analyzer (Malvern, Worcestershire, UK [9]. All the samples were prepared with the following procedure. The polymer powder was redispersed into water with a solid content of 30%. The sample was measured after dilution with deionized water, to obtain clarity, and 30 min of ultrasonication. Each was determined three times, then the average experimental result was recorded.

#### 2.3.3. Zeta Potential of the Latex Powder

Zeta potential for the sample was calculated via ZetasizerNano analyzer (Malvern, Worcestershire, UK). The sample was the same as discussed in Section 2.3.2.

### 2.4. Measurements of Cement Paste with Latex

**Figure 2:**
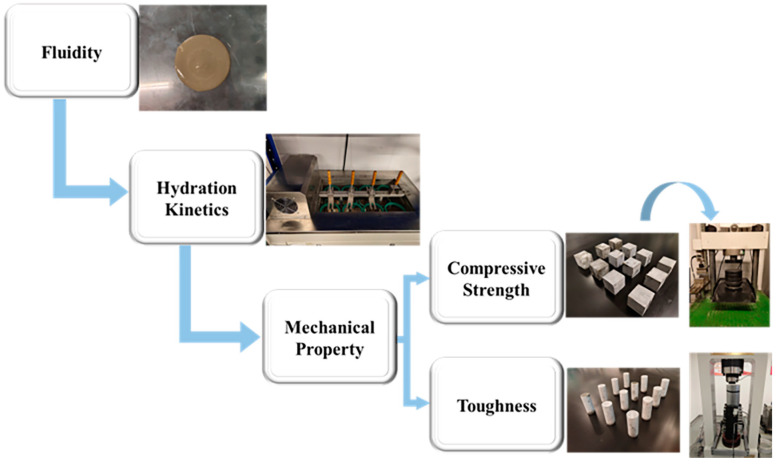
Experiments with modified cement.

The fluidity, isothermal heat flow calorimetry, triaxial mechanical testing, and compressive strength measurements were performed on latex modified cement, as illustrated in Figure 2.

#### 2.4.1. Fluidity of Cement Paste

The preparation of cement slurry was conducted following the procedure mentioned in standard GB/T19139-2012, “Testing of well cements”, and the water-to-cement ratio was 0.44, in terms of the ISO 10426-1:2009 standards and API Recommended Practice 10B and 10B2. The dosage of latex powder was 1.2 wt% (according to cement), which was redispersed evenly in water before mixing with the cement. The fluidities of the cement pastes after standing for 1 min, 15 min, 30 min, 45 min, 60 min, 90 min, and 120 min were determined. The fluidity of cement slurry was determined via a mini-cone, which was 60 mm high, with a bottom diameter of 60 mm and top diameter of 36 mm, following the standard GB/T 2419–2005. The cement slurries were poured in the cone and lifted immediately. After the cement slurry has been flowing for 30 s, its horizontal and vertical spread diameters were recorded; the average value was then calculated.

A mixture of 20 g cement, 0.48 g latex powder and tap water (where the water–cement ratio was 0.44) was diluted 50 times and then sampled and observed through an optical microscope.

#### 2.4.2. Isothermal Heat Flow Calorimetry

The retardation effect of SISBR latex powder on the hydration process was studied via isothermal calorimetry and was found to be an effective method for monitoring cement hydration [7]. An isothermal calorimeter (Yite, Wuhan, China) was applied to track the hydration process. The homogeneously mixed cement slurry was immediately poured in the isothermal calorimeter channel to investigate the cement hydration. The heat flow curves of samples were plotted at 60 °C for 96 h.

#### 2.4.3. Triaxial Mechanical Test

The triaxial mechanical test was conducted with the standard SY/T 6466—2016. Using a triaxial mechanical testing machine (MOOG, New York, NY, USA), the triaxial stress–strain curves of 2.4% SISBR latex modified cement, and pure cement curing at 60 °C for 7 d were measured. The confining pressure used in the test was 20 MPa.

#### 2.4.4. Compressive Strength

The compressive strength tests of cement samples (50.8 × 50.8 × 50.8 mm) containing 1.2% SISBR latex was conducted using a testing machine (Naier, Jinan, China) after curing for 7 days at 60 °C. The loading speed utilized was 1.2 kN/s.

### 2.5. Reaction Mechanism of Latex Powder

#### 2.5.1. X-ray Diffraction Tests

The modified cement stone was characterized by XRD and the cement hydration was tracked according to the structure of the hydrated product. The cement cubes were ground, then soaked in absolute ethanol for 24 h, followed by drying in preparation for XRD. The Bruker D8 Advance X-ray diffractometer (Bruker, Karlsruhe, Germany) was utilized to carry out the measurements from 5 to 70° at a speed of 7°/min [17,18].

#### 2.5.2. Adsorption Measurement

Adsorption ability of the polymer latex onto the mineral surface was determined via the total organic carbon apparatus (Shimadzu, Kyoto, Japan). The water-to-cement ratio was 100:1. The fresh cement with SISBR latex was continuously stirred for 2 h. The sample was centrifuged at a speed of 10,000 r/min for 10 min to obtain the supernate, and then it was passed through the syringe filter (0.45 mm). A TOC analyzer was used to determine the amount of organic carbon in the dilute solution. The amount of latex adsorbed onto cement could be obtained via reducing the amount of latex contained in the supernate from that in the initial solution.

#### 2.5.3. Scanning Electron Microscope (SEM) Tests

The micro-morphology of cement stone was observed by scanning electronic microscope ( Hitachi, Tokyo, Japan) to characterize hydration products. Before the SEM tests, the samples were coated with gold under vacuum condition.

## 3. Results and Discussion

### 3.1. Dispersion State Analysis of SISBR Latex Powder

**Figure 3:**
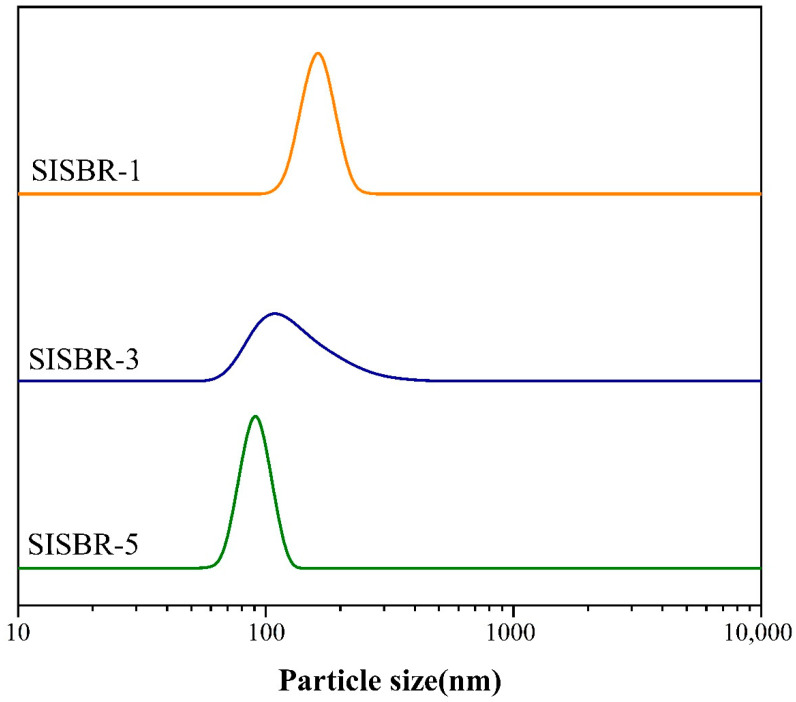
Effects of IA amount on particle size of SISBR latex.

To study the dispersion state of latex accurately, we tested particle sizes of latex samples [19]. Figure 3 presented the particle size distribution of SISBR latex with varied addition of IA. The addition of IA in the three latex samples reached 1 wt%, 3 wt%, and 5 wt%, and the particle sizes of dispersed emulsions were 128 nm, 106 nm, and 96 nm, respectively. The particle size of latex dispersed in water decreased significantly with the increase in IA amount, which demonstrated that the increased amount of IA in SISBR could inhibit the agglomeration of latex powder in water.

**Figure 4:**
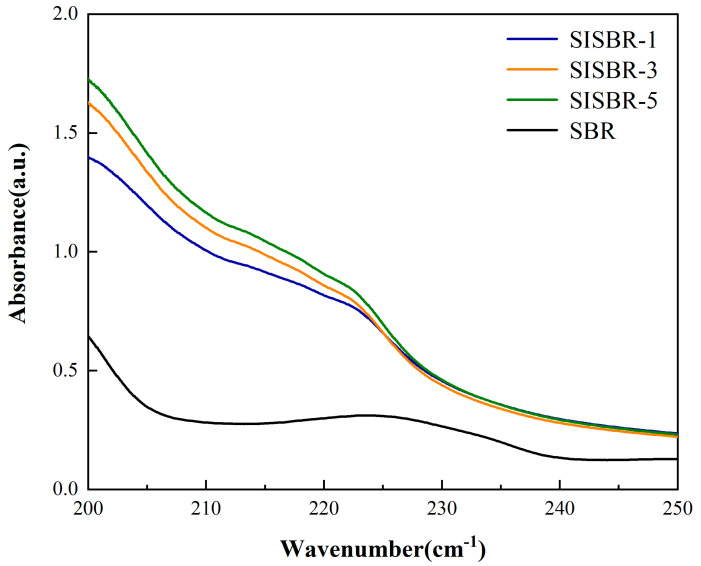
The correlation between the absorbance of latex and IA amount.

In this part, the absorbance of redispersed latex was adopted to evaluate the dispersing ability of latex powders. According to Figure 4, the absorbance of SBR latex was significantly lower than that of SISBR samples in all wavelengths, which showed the aggregation and sedimentation of SBR latex powder in water. The absorbances of SISBR latex samples increased with the increase in the IA percentage, which demonstrated that the latex with more IA showed more effective dispersion. This was because the hydrophilic carboxyl (-COOH) groups in IA could facilitate the dispersion of latex powder in water, so the rising amount of -COOH would improve the dispersion stability [20].

**Figure 5:**
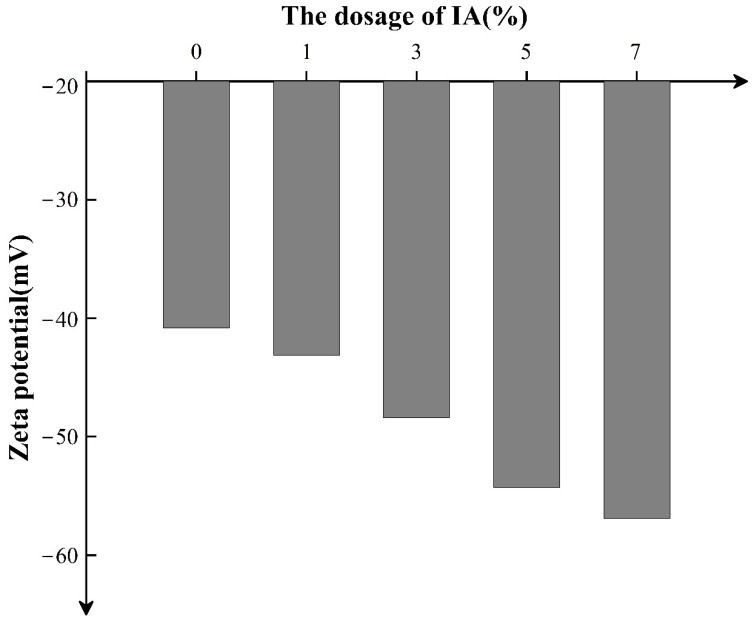
Zeta potential of SISBR latex at various dosages of IA.

The dispersion state of latex relates to the electrostatic repulsion. Zeta potential has been reported to correspond to the electrostatic repulsion force among particles in solution, and higher absolute zeta potential demonstrates stronger electrostatic repulsion among particles. To quantitatively analyze the dispersion stability of latex powder, zeta potentials of latex powder samples with different IA amounts were measured through dynamic light scattering. In Figure 5, the latex without IA exhibited lower absolute zeta potential (40.83 mV) under the weaker electrical repulsion among the latex grains. The addition of IA in latex substantially improved zeta potential, and the absolute potential value was further improved with the increase in the added amount of IA. With the IA amount increasing to 7 wt%, the absolute zeta potential of latex aqueous solution reached 56.9 mV. The increased zeta potential of the SISBR sample is induced by the negative charge on the latex particle surface, which is correlated with the amount of carboxylic ion, depending on the IA amount. The negative charges of carboxylic ion originated from IA distributed on surfaces of latex grains adequately, which formed the electric double layer in the water. The elevation in IA amount increased the negative charge of latex and thickened the electric double layer, inducing a strong electrostatic repulsion force, which led to high stability of the latex [21]. Thus, the absolute zeta potential of latex improved with the increasing IA dosage. On the whole, when the absolute zeta potential exceeded 40 mV, the dispersion was stable [22]; otherwise, the latex grains aggregated and exhibited poor stability. Thus, the SISBR with higher absolute zeta potential dispersed better than the SBR latex.

### 3.2. Influence of SISBR Latex on Cement Slurry

#### 3.2.1. Fluidity

**Figure 6:**
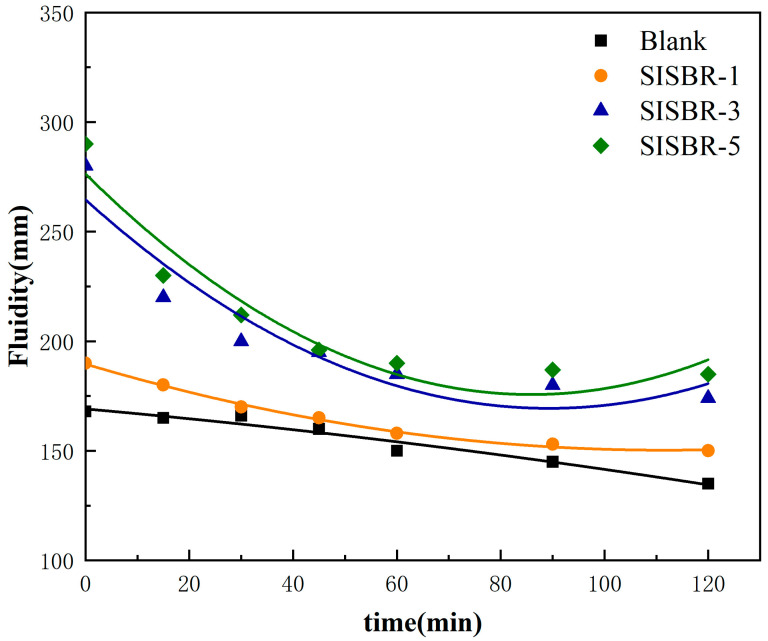
Fluidity behavior of the cement pastes with SISBR latex.

Figure 6 presents the fluidities of latex-modified cement pastes. The sample with SISBR-1 latex exhibited a lower fluidity (<200 mm), which is difficult to pump. The effects exerted by SISBR-3 latex and SISBR-5 latex on the cement fluidity were consistent, and their initial fluidities reached 283 mm and 290 mm, respectively, largely greater than the blank cement sample. The fluidities of cement pastes with SISBR-3 and SISBR-5 notably decreased first and then slightly increased over time. The fluidity of cement increased with increasing IA content in SISBR latex, and the cement paste became thinner and showed higher workability, from the visual observation [4]. This was because the -COOH groups from IA, which has a higher adsorption capability [7], promoted the adsorption of latex onto cement particles. As anchoring groups, carboxyl groups of latex provided electrostatic repulsion, and inhibited the agglomeration of cement particles [23,24]. Meanwhile, the benzene ring, as a rigid chain, could provide steric hindrance for cement grains and enhance its fluidity [24], which demonstrated that SISBR latex had a water-reducing effect. Additionally, it has been reported that the improved fluidity can also be ascribed to the air-entrainment effect and ball-bearing action of polymer particles at nanoscale, which reduced flow resistance of cement grains [23].

#### 3.2.2. Optical Microstructure of Cement Slurry

**Figure 7:**
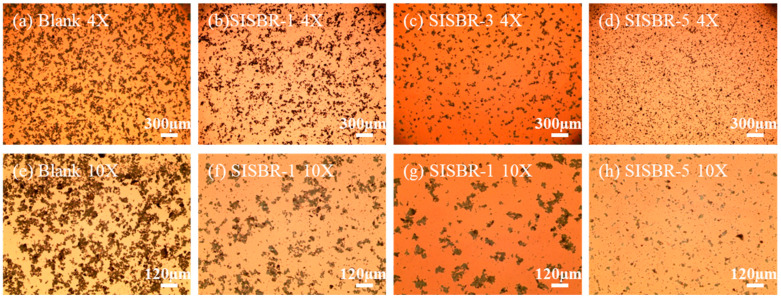
Microstructure of cement paste; pure cement in 4X (**a**) and 10X (**e**), SISBR-1 sample in 4X (**b**) and 10X (**f**), SISBR-3 sample in 4X (**c**) and 10X (**g**), SISBR-5 sample in 4X (**d**) and 10X (**h**).

An optical microscope was used to examine the dispersing state of cement particles with latex powder [25,26]. Figure 7a–d presents the microscope images of pure cement paste magnified four times, cement paste with SISBR-1 latex, SISBR-3 latex, and SISBR-5 latex, respectively. Figure 7e–h presents the microscope images magnified ten times.

As clearly indicated from the figures, there were considerable flocculation structures with different sizes for the pure cement paste (Figure 7a), due to the attraction of opposite charge generated by cement hydration and van der Waals force between cement particles. The flocculation structure was broken by adding SISBR latex samples. Furthermore, the sizes of cement aggregates decreased with increased IA in latex, especially for SISBR-5 latex with the smallest flocculent structure (Figure 7d,h), which demonstrated an improved dispersed state. These images also demonstrated that the addition of IA in latex improved the dispersion state of cement slurry, which can also support the fluidity results in Section 3.2.1.

**Figure 8:**
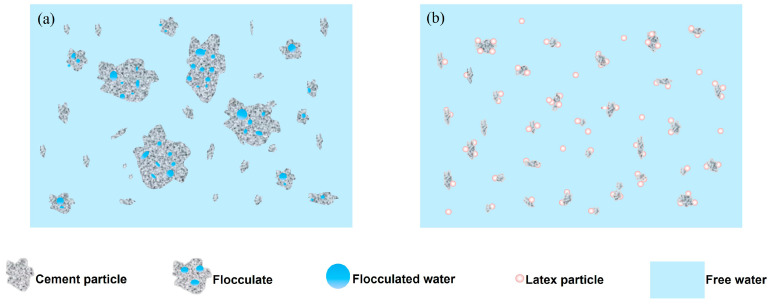
Schematic diagram of the influence of SISBR latex on cement paste; cement flocculation (**a**), the latex disassembled the cement flocculates (**b**).

The dispersion mechanism was interpreted in Figure 8. The flocculation between colloidal structure could be well explained using the Derjaguin–Landau–Verwey–Overbeek (DLVO) theory, that is, the competition between van der Waals force and electric double layer force. As shown in Figure 8a, the cement flocculates exhibited a continuous grid structure mainly formed by van der Waals forces among cement grains, and the reduced water content of the dispersed phase decreased the fluidity of the pure cement. SISBR latex adsorbed onto cement served as the physical isolation to split the cement grains by weakened van der Waals forces. Meanwhile, the latex enhanced the electrostatic repulsion interaction among the grains and disassembled the cement flocculates (Figure 8b) [27,28]. According to the results in Figure 5, the increased surface potential enhances the electrostatic repulsion potential energy with the increasing IA amount in latex, and the level of potential energy barrier of total potential energy curve increases, which finally prevents the agglomeration of cement particles [29].

### 3.3. Effects of SISBR Latex Powders on Cement Hydration

#### 3.3.1. Hydration Kinetics

**Figure 9:**
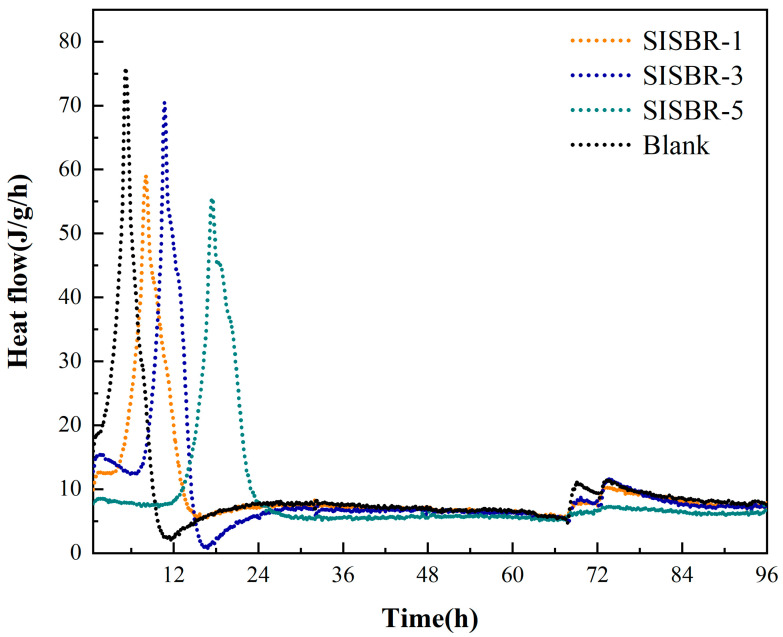
Influences of IA amount of SISBR latex powders on heat evolution of cement hydration.

The effects of various IA amounts of latex on the differential heat flow of slurry are shown in Figure 9. The hydration peak of the acceleration period shifted to the right continuously, and the time to reach the maximal exothermic rate was prolonged with more IA added in the latex powder. The retarded hydration process was mainly the result of the chelation of calcium ions (Ca^2+^) and -COOH in latex, which decreased the concentration of Ca^2+^ in pore solution and inhibited the formation of cement hydrates [30,31]. Meanwhile, the adsorbed latex could form a covering layer, which would occupy the reactive sites and restrain the spread of ions and water on the interface between aqueous phase and cement phase. Following the adsorption and chelation processes, SISBR-5 latex with the most -COOH groups induced the longest induction period. Moreover, not merely the amount of -COOH groups but the separation distance of the anionic groups also greatly affected its chelation with Ca^2+^ [32]. As a result, the retardation effect of the cement hydraulic reaction was stronger with increasing dosage of IA, on account of the higher density of -COOH groups along the polymer backbone.

#### 3.3.2. X-ray Diffraction Analysis

**Figure 10:**
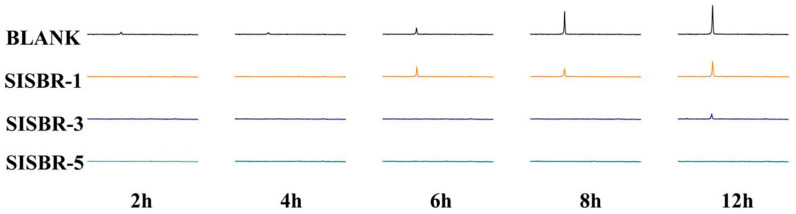
Time-dependent evolution of the CH signal (2θ = 18.01°) of the hydrating cement pastes with SISBR latex powder measured via XRD.

To study the retardation effects of latex powder more specifically on cement hydration, XRD spectra of cement hydrate at the early age was measured. During hydration, calcium hydroxide (CH) was one of the major crystalline products, which showed a spike at 2θ = 18.01°. And its emergence time would refer to the extent of cement hydration [33]. Figure 10 summarizes the accurate time-dependent evolution of CH in the cement hydration with SISBR-1, SISBR-3, and SISBR-5 latex, respectively. As indicated from the figure, the addition of the three latex samples delayed the emergence time of CH to varying degrees. For cement paste with SISBR-1 latex, CH crystallization took place at 6 h after mixing with water, consistent with the blank sample, but the peak intensity decreased. The SISBR-3 sample was extended to 12 h. For SISBR-5 latex-modified cement paste, CH formation was almost overall suppressed, and no CH crystallization was performed in 12 h, which significantly hindered the development of cement microstructure. The stronger retarding effect demonstrated that the -COOH resulting from IA acted as a main factor to inhibit the formation of CH [34].

#### 3.3.3. Adsorption Behavior of SISBR Latex Powders on Cement Surface

**Figure 11:**
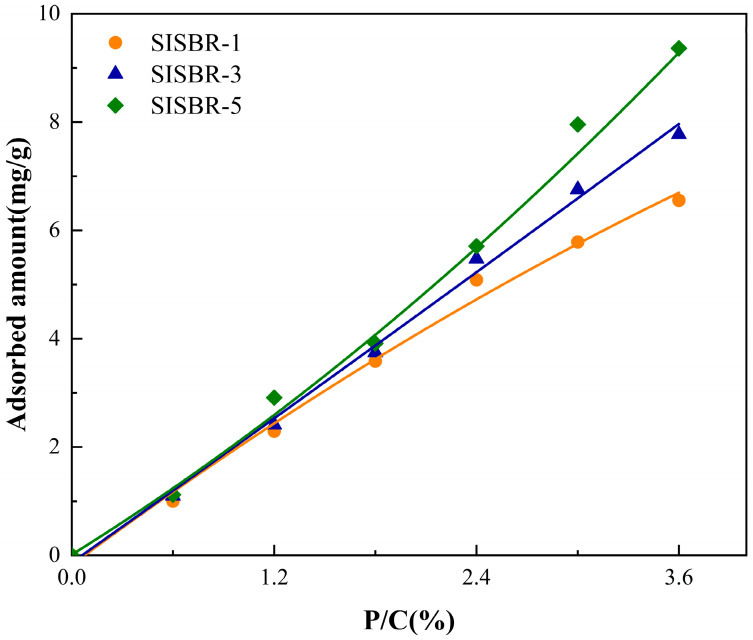
Adsorption behavior of SISBR latex on the surface of cement particles.

To further verify the retarding mechanism of SISBR latex on the hydration process, the adsorption of SISBR latex powders containing varied IA amounts on cement particles was measured via adsorption isotherm. In Figure 11, the adsorption performance of latex powder was improved with added IA. It has been proved that with the irregularly distributed charge on cement particle surfaces, the charged latex can be greatly adsorbed onto the cement or hydration products, such as ettringite, due to the attraction of opposite charges [35,36]. Furthermore, the chelation effect between the latex and Ca^2+^ attached onto the negatively charged cement surfaces also facilitated the adsorption [37,38]. The amount of -COOH usually determines the adsorption performance of latex owing to the electrostatic attraction and chelation between cement and latex. Thus, the increasing IA enhanced the adsorption of latex onto the cement grain, preventing contact between water and the cement particles [39].

### 3.4. Effects of SISBR Latex on Mechanical Property of Cement

#### 3.4.1. Effects of SISBR Latex on Compressive Strength of Cement

**Figure 12:**
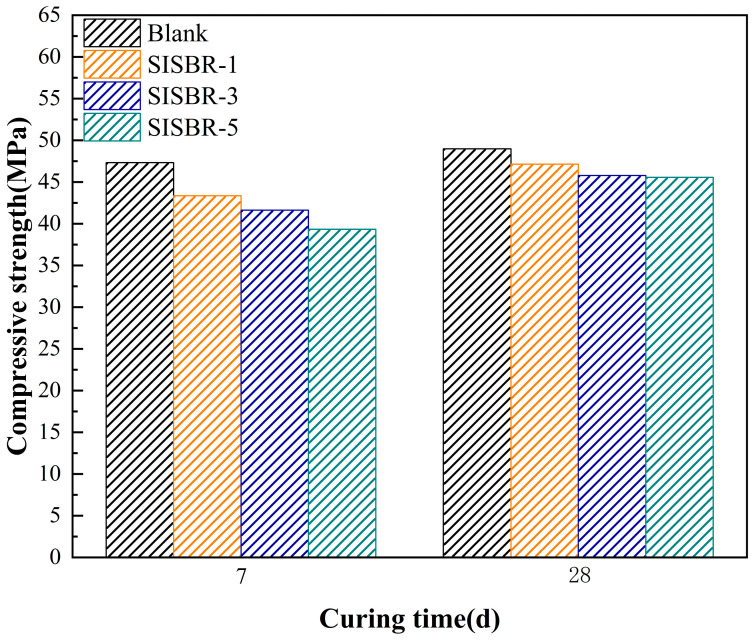
Compressive strength of hardened cement with latex.

Compressive strength measurements of cement stones with various latex samples (SISBR-1, SISBR-3, and SISBR-5) under different aging times are indicated in Figure 12. The compressive strength of cement decreased with the increasing IA of latex. The greatly adsorbed latex particles could form the covering layer on the surfaces of cement particles that inhibited the nucleation reactive process of hydrated products by occupying the reactive sites, such as Hydrate calcium silicate (CSH), owing to a higher interfacial energy between the CSH and polymer phases [40]. Thus, SISBR latex may form the polymer film, covering part of the cement grains and hydration products and hampering ion diffusion, then retarding the cement hydration. Some studies have reported that the air entrapment in SBR-modified cement, which also displayed large porosity, resulted in the decreasement of compressive strength [41]. Since round and smooth SISBR latex entrapped air during the mixing process and produced harmful air bubbles in the stone, this characteristic contributed to the decreased compressive strength.

#### 3.4.2. Effects of SISBR Latex on Toughness of Cement

**Figure 13:**
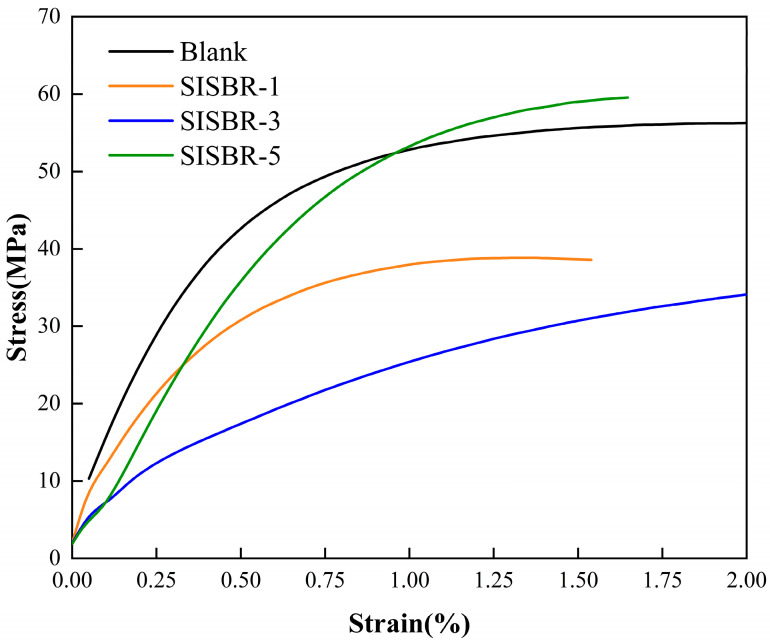
Stress–strain curve of cement paste with SISBR latex powder.

Due to the brittle nature of cementitious materials, cement sheath is easy to crack when impacted by extra load and environmental exposure. By adding latex, the toughness of cement cubes can be improved. Figure 13 shows the triaxial stress–strain curves of three latex-modified samples and a blank sample. As revealed, the cement cubes in the presence and absence of SISBR latex exhibited elastic properties under the initial stress. However, with the increase in load, the stress–strain relationship slowly displayed a change in nonlinearity; the cement stone changed to ever-increasing plastic deformation under the high-pressure load. In contrast to the blank sample, SISBR-3 latex-modified cement stone exhibited significantly better plastic deformation, which probably contributed to its good dispersion and adsorption in cement. The well-dispersed SISBR-3 could form a uniform polymer film [42] in hydration products. The continuous polymer film within the cement could absorb part of the applied force during the brittle fracture process, boosting the degree of toughness and compactness. Moreover, the latex particles could fill up the inner macroscopic and microscopic defects of the cement stone.

As revealed from the slope of the stress–strain curve in elastic deformation (shown in Table 2), the elastic modulus first decreased and then increased with the addition of IA. The increasing elastic modulus of SISBR-5 latex-modified cement, was attributed to the over-retardation of the hydration progress. In brief, the SISBR-3 latex dramatically reinforced the toughness of the cement matrix, improving the ability to resist crushing impact.

#### 3.4.3. Effects of SISBR Latex on Microstructure of Cement Stone

**Figure 14:**
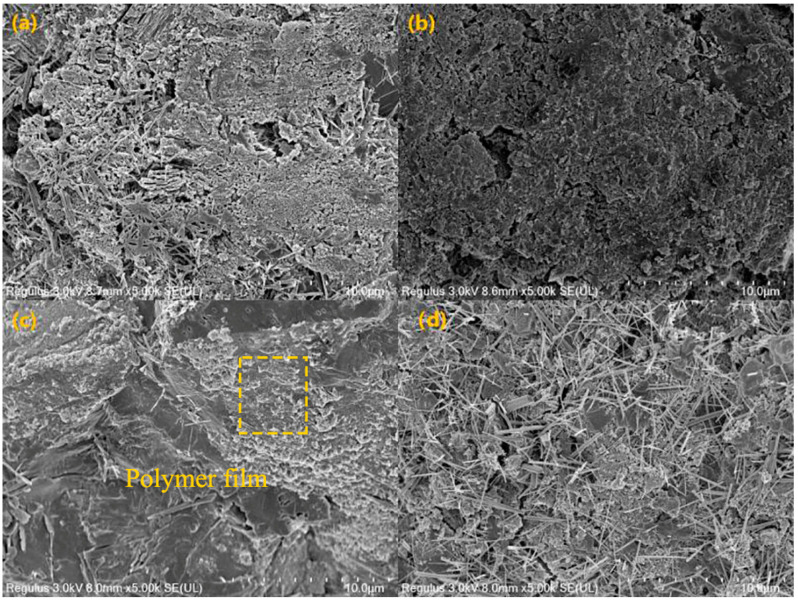
SEM images of hydrated products with SISBR latex powders. (**a**) Pure cement, (**b**) SISBR-1 latex cement sample, (**c**) SISBR-3 latex cement sample, (**d**) SISBR-5 latex cement sample.

The effects of three latex samples on the microstructure of the cement cubes cured for 7 d were examined. In Figure 14a, hydration products of the blank sample were loosely packed with considerably large voids. Figure 14b illustrates the microstructure of hardened cement containing SISBR-1 latex. The SISBR-1 latex could fill the pores among hydration products and produce more compact microstructure to dissipate external mechanical force. Figure 14c presents the SEM image of the SISBR-3 modified cement sample. The latex film covered the surface of hydrated product and bound it firmly together, filling the voids and cracks in the stone, which enhanced the toughness through assuming part of the energy. As can be seen in Figure 14d, numerous harmful pores and needle-like, inter-growing ettringite (AFt) crystals emerged in the sample modified by SISBR-5 latex [43,44] due to the significant retardation exerted by IA on cement hydration. Accordingly, SISBR-3 is more effective for improving the toughness of cement.

## 4. Conclusions

To solve the problem of liquid latex in transportation and storage, the SISBR-3 latex powders with an average particle size of 106 nm were successfully prepared in this study, which largely improved the toughening efficiency. The following are the main conclusions of this study:

The dispersion ability, the key property of latex powder, was enhanced with the increasing-COOH groups in SISBR, which could facilitate the electrostatic repulsion among latex particles. Based on this, through the systematic study for hydration kinetics and toughening mechanism of latex-modified cement, the SISBR-3 was verified to be the promising candidate to replace liquid latex with the best toughening efficiency. The increasing IA in latex will retard the hydration process of cement due to the excessive adsorption amount on cement grains and stronger chelation with Ca^2+^. Thus, the cement modified by SISBR exhibited decreased compressive strength. Additionally, the sample with SISBR-5 displayed a porous microstructure and less toughness due to the over-retardation in cement hydration. The SISBR-3 resulted in a decrement of 62% in the elastic modulus compared with the plain cement and greatly improved the toughness of the cement. This was due to the polymer film formed among hydration products, which can greatly dissipate external mechanical force. While the elastic modulus of SISBR-5 latex- reinforced cement increased to 8.4 GPa, due to the over-retardation in hydration progress.

This approach provides a cost-effective and efficient design for SISBR latex storage and transport to support large-scale industrial application. The excellent toughening efficiency of SISBR-3 on cement can ensure the integrity of cement sheath in complicated conditions. Future experimentation can further explore the widespread usage of latex powder in cementitious materials.

## Figures and Tables

**Table 1:** Chemical composition of G class oil well cement.

Chemical Compositions (wt.%)							
CaO	SiO_2_	Fe_2_O_3_	SO_3_	Al_2_O_3_	MgO	K_2_O	Others
69.68	15.13	6.45	3.39	2.34	0.92	0.79	1.3

**Table 2:** Comparison of elastic modulus.

Latex Samples	IA Dosage (%) in Latex	Elastic Modulus (GPa)
SBR	0	8.34
SISBR-1	1	5.9
SISBR-3	3	3.16
SISBR-5	5	8.4

## Data Availability

Data are available on request.
